# Safety of immune checkpoint inhibitors in non-small-cell lung cancer patients with idiopathic interstitial pneumonia: a matched case–control study

**DOI:** 10.1007/s00280-021-04362-7

**Published:** 2021-10-14

**Authors:** Takenori Ichimura, Miwa Hinata, Daisuke Ichikura, Shinya Suzuki

**Affiliations:** 1 Department of Hospital Pharmaceutics, School of Pharmacy, Showa University, 1-5-8 Hatanodai, Shinagawa-ku, Tokyo, 142-8555 Japan; 2 Department of Pharmacy Services, Showa University Northern Yokohama Hospital, Yokohama, Kanagawa Japan

**Keywords:** Interstitial pneumonia, Immune checkpoint inhibitor, Non-small-cell lung cancer, A case–control study

## Abstract

**Purpose:**

The immune checkpoint inhibitor nivolumab is commonly used for non-small-cell lung cancer treatment. Immune checkpoint inhibitors cause immune-related adverse events, including interstitial pneumonia. However, there are no studies on the risk factors for interstitial pneumonia exacerbation after immune checkpoint inhibitor administration in patients with a history of different types of interstitial pneumonia. Therefore, we aimed to investigate the risk factors for interstitial pneumonia exacerbation in patients with non-small-cell lung cancer and a history of interstitial pneumonia. We also aimed to explore differences in the risk of interstitial pneumonia exacerbation due to various types of interstitial pneumonia—idiopathic interstitial pneumonia, immune-related pneumonitis, and radiation pneumonitis.

**Methods:**

Eleven patients with a history of interstitial pneumonia exacerbation following the administration of immune checkpoint inhibitor were included in the study. We performed 1:2 matching based on age and sex. Twenty-two patients whose interstitial pneumonia did not worsen after immune checkpoint inhibitor administration belonged to the control group. We calculated odds ratios for each factor in the patients and control subjects.

**Results:**

The odds ratio of idiopathic interstitial pneumonia in the case group was 0.15 (95% confidence interval: 0.03–0.89) (*p* = 0.03). There were no significant differences in other factors, such as smoking history, pulmonary emphysema, and chronic obstructive pulmonary disease.

**Conclusion:**

The administration of immune checkpoint inhibitors in non-small-cell lung cancer patients with a history of idiopathic interstitial pneumonia might be a viable treatment option and have clinical benefits.

## Introduction

Immune checkpoint inhibitors (ICIs) are an important component in the treatment of non-small-cell lung cancer (NSCLC) [1]. The ICI nivolumab was first used in treating NSCLC, followed by pembrolizumab, atezolizumab, and durvalumab [2]. In Japan, ICIs are recommended as the first-line treatment for this disease if there are no gene mutations [3].

ICIs reportedly cause immune-related adverse events, including interstitial pneumonia [4]. The incidence of interstitial pneumonia in NSCLC patients in a phase III trial was 3.8% (*n* = 287) with nivolumab [5], 5.8% (*n* = 154) with pembrolizumab [6], and 1.6% (*n* = 609) with atezolizumab [7]. A meta-analysis of systematic reviews has also reported the incidence, diagnosis, risk factors, and management of immune-related interstitial pneumonia [8, 9]. Interstitial pneumonia can lead to serious complications, including death. Therefore, depending on the severity, the drug might be withdrawn or discontinued when interstitial pneumonia develops after the administration of ICIs [10].

A history of interstitial pneumonia is a risk factor for the development of ICI-induced interstitial pneumonia. For example, after the administration of atezolizumab to 17 patients with NSCLC and a history of idiopathic interstitial pneumonia, 5 (29.4%) had exacerbated interstitial pneumonia [11]. Furthermore, after the administration of nivolumab in 2 independent studies, 2 of 18 (11.1%) [12] and 8 of 26 (30.8%) [13] patients with NSCLC and a history of idiopathic interstitial pneumonia showed worsening of ICI-induced interstitial pneumonia.

However, these were reports on a single group without comparison among patients with NSCLC with a history of idiopathic interstitial pneumonia.

Based on these reports, a history of interstitial pneumonia is considered a risk factor for development of interstitial pneumonia following the administration of ICIs. Therefore, it is necessary to consider whether it is appropriate to administer ICIs to patients with a history of interstitial pneumonia [14]. Accordingly, in Japan, the Ministry of Health, Labor, and Welfare, which controls the approval and proper use of medicines, has warned against administering ICIs to patients with a history of interstitial pneumonia, regardless of the type of interstitial pneumonia.

However, differences in the risk of exacerbation of interstitial pneumonia due to various types of interstitial pneumonia, such as idiopathic interstitial pneumonia, immune-related pneumonitis, and radiation pneumonia, are unknown. It has also been reported that the risk factors for the development of interstitial pneumonia after administration of ICIs in the absence of a history of interstitial pneumonia include a history of respiratory disease, radiation therapy, and smoking [15]. Additionally, it has been pointed out that male sex, smoking history, and combination therapy (including ICI and cytotoxic drugs) are potential risk factors for interstitial pneumonia [8, 16]. However, other risk factors in patients with a history of this interstitial pneumonia are unknown. A history of interstitial pneumonia is a risk factor for recurrent interstitial pneumonia due to ICI administration. However, ICIs are also an important option for the treatment of NSCLC [1]. Therefore, despite a history of interstitial pneumonia, if the benefits of treatment outweigh the risks of interstitial pneumonia exacerbation, ICIs might be administered. Thus, identifying risk factors other than a history of interstitial pneumonia can lead to optimal treatment selection.

This case–control study aimed to clarify risk factors of exacerbating interstitial pneumonia after the administration of ICIs in patients with a history of interstitial pneumonia. We further aimed to investigate the differential risk of interstitial pneumonia exacerbation due to various interstitial pneumonia types (i.e., idiopathic interstitial pneumonia, immune-related pneumonitis, and radiation pneumonitis).

## Patients and methods

### Patients

The research facility was located at the Showa University Northern Yokohama Hospital in Japan. The study period was conducted from December 2015 to March 2020. Among NSCLC patients who received monotherapy or combination therapy with nivolumab, pembrolizumab, and atezolizumab, inclusion criteria were KL-6 (Krebs von den Lungen 6 glycoprotein) ≥ 500 U/mL or SP-D (Surfactant protein D) ≥ 110 ng/mL and an ICD10 (International Classification of Diseases, 10th revision, Clinical Modification) of interstitial lung disease (J849), resulting in 183 patients registering for the study.

### Study design

We conducted a case–control study. Among the 183 patients with NSCLC and a history of interstitial pneumonia, 11 had exacerbation of interstitial pneumonia after administration of ICIs. Eleven patients in the case group whose interstitial pneumonia worsened after administration of ICIs were matched by age (± 5 years) and sex. As a result, 22 subjects in the control group whose interstitial pneumonia did not worsen were enrolled as controls (1:2 matching).

### Study assessment

The primary objective of this case–control study was to identify risk factors. We investigated the following: drugs, smoking history, medical history, surgical history, staging, distant metastasis, regimen and number of treatments, pretreatment history, survival time, treatment period with anticancer drugs, and treatment regimen after exacerbation of interstitial pneumonia.

The secondary objective of this case–control study was to elucidate the differential risks of exacerbation of interstitial pneumonia among different types of interstitial pneumonia (i.e., idiopathic interstitial pneumonia, immune-related pneumonitis, and radiation pneumonitis).

In addition to these two objectives, we also investigated the duration of therapy and overall survival. Patients with untraceable treatment or survival were investigated until the date of traceability.

As previously described [9], when clinical symptoms such as cough, dyspnea, and fever were present upon examination, along with elevated KL-6 and SP-D levels, doctors suspected interstitial pneumonia and performed computed tomography (CT). The respiratory physician examined the CT and identified characteristics of immune-related pneumonia during ICI administration {mainly patterns of organizing pneumonia, nonspecific interstitial pneumonia, hypersensitivity pneumonia, acute interstitial pneumonia, and respiratory bronchiolitis-associated interstitial pneumonia [10]}, and made the diagnosis. Interstitial pneumonia was comprehensively diagnosed based on clinical, physiological, and chest CT findings. Respiratory physicians diagnosed the need for inpatient treatment. The severity was based on the term “pneumonitis” in the common terminology criteria for adverse events (CTCAE) version 5. In this study, the terms were used as follows. ICI-induced interstitial pneumonia was used as a synonym for ICI-induced pneumonitis. Radiation pneumonitis was also considered as a subclass of interstitial pneumonia.

### Statistical analysis

Continuous variables were compared using a two-sided *t* test or the Mann–Whitney *U* test. Categorical variables were compared using the chi-square test or Fisher’s exact test.

For the case and control groups, we calculated the odds ratio and 95% confidence interval (CI) for a risk factor to be present. The JMP^®^ Pro 15.0.0 software (SAS Institute Inc., Cary, NC, USA) was used for statistical analysis, and the significance level was set at *p* < 0.05. There were no missing data for the evaluation objectives.

## Results

### Characteristics

Patient backgrounds in the case and control groups are shown in Table 1. The average age was 69.1 years, and the proportions of males and females—90.9% and 9.1%, respectively. Except for the history of interstitial pneumonia (idiopathic interstitial pneumonia), there was no significant difference between the two groups.

**Table 1:** Clinical patient characteristics

Characteristics	Case group (*n* = 11) *n* (%)	Control group (*n* = 22) *n* (%)	*p* value
Age			
Average	69.1	69.1	
Median (range)	71 (50–81)	71 (51–86)	
Sex			
Male	10 (90.9)	20 (90.9)	
Female	1 (9.1)	2 (9.1)	
Smoking history			
Current	1 (9.1)	2 (9.1)	1.00
Past	10 (90.9)	17 (77.3)	0.64
Never	0	3 (13.6)	0.53
Histology			
Adenocarcinoma	7 (63.6)	17 (77.3)	0.44
Squamous cell carcinoma	4 (36.4)	4 (18.2)	0.39
Not otherwise specified	0	1 (4.5)	1.00
PD-L1 expression [TPS (%)]			
<1%	0	4 (18.2)	0.28
1–49%	2 (18.2)	4 (18.2)	1.00
≥50%	3 (27.3)	5 (22.7)	1.00
Unknown	6 (54.5)	9 (40.9)	0.49
Stage			
III	4 (36.4)	3 (13.6)	0.19
IV	7 (63.6)	19 (86.4)	0.19
History of lung surgery	3 (27.3)	4 (18.2)	0.66
History of radiation therapy	6 (54.5)	8 (36.4)	0.46
History of illness			
Pulmonary emphysema and COPD	1 (9.1)	2 (9.1)	1.00
Treatment line			
1	2 (18.2)	6 (27.3)	0.69
2	4 (36.4)	2 (9.1)	0.15
3	2 (18.2)	8 (36.4)	0.43
≥4	3 (27.3)	6 (27.3)	1.00
ICI			
Nivolumab	7 (63.6)	11 (50.0)	0.71
Pembrolizumab	3 (27.3)	9 (40.9)	0.70
Atezolizumab	1 (9.1)	2 (9.1)	1.00
History of interstitial pneumonia			
Radiation pneumonitis	5 (45.5)	4 (18.2)	0.12
ICI-induced interstitial pneumonia	4 (36.4)	5 (22.7)	0.43
Idiopathic interstitial pneumonia^a^	2 (18.2)	13 (59.1)	0.03

*PD-L1* programmed death-ligand 1; *TPS* tumor proportion score; *COPD* chronic obstructive pulmonary disease; *ICI* immune checkpoint inhibitors

^a^Idiopathic interstitial pneumonia is an interstitial pneumonia of unknown cause

### Risk factors

We tested each factor between the two groups (Table 2). The odds ratio for idiopathic interstitial pneumonia in the case group relative to that in the control group showed a significant difference at 0.15 (95% CI 0.03–0.89) (*p* = 0.03). There were no significant differences in other factors, such as smoking history, pulmonary emphysema, and chronic obstructive pulmonary disease, between the two groups.

**Table 2:** Risk factors for interstitial pneumonia

Risk factor	Case group (*n* = 11) *n* (%)	Control group (*n* = 22) *n* (%)	*p* value
Smoking history			
Current and past	11 (100)	19 (86.4)	0.54
Never	0	3 (13.6)	
Histology(except NOS)			
Adenocarcinoma	7 (63.6)	17 (81.0)	0.40
Squamous cell carcinoma	4 (36.4)	4 (19.0)	
PD-L1 expression [TPS (%)] (except unknown)			
<1%	0	4 (30.8)	0.28
≥1%	5 (100)	9 (69.2)	
Stage			
III	4 (36.4)	3 (13.6)	0.19
IV	7 (63.6)	19 (86.4)	
History of lung surgery			
Yes	3 (27.3)	4 (18.2)	0.66
No	8 (72.7)	18 (81.8)	
History of radiation therapy			
Yes	6 (54.5)	8 (36.4)	0.46
No	5 (45.5)	14 (63.6)	
History of illness: pulmonary emphysema and COPD			
Yes	1 (9.1)	2 (9.1)	1.00
No	10 (90.9)	20 (90.9)	
Treatment line			
1	2 (18.2)	6 (27.3)	0.69
≥2	9 (81.8)	16 (72.7)	
History of interstitial pneumonia			
NSCLC treatment-related interstitial pneumonia^a^	9 (81.8)	9 (40.9)	0.03
Idiopathic interstitial pneumonia^b^	2 (18.2)	13 (59.1)	

*NOS* not otherwise specified; *PD-L1* programmed death-ligand 1; *TPS* tumor proportion score; *COPD* chronic obstructive pulmonary disease; *NSCLC* non-small-cell lung cancer

^a^ICI-induced interstitial pneumonia and radiation pneumonitis

^b^Idiopathic interstitial pneumonia is an interstitial pneumonia of unknown cause

### Duration of therapy and overall survival

The duration of treatment and overall survival are shown in Table 3. The median treatment period in the case group (except for three survivors) was 588 days and that in the control group (except for five survivors) was 450 days. The median overall survivals in the case and control groups were 661 and 506 days, respectively.

**Table 3:** Duration of therapy and overall survival

	Case group except 3 survivors (*n* = 8)	Control group except 5 survivors (*n* = 17)
Duration of therapy with anticancer drugs (days) (except survivors)		
Median (range)	588 (98–1504)	450 (90–2098)
Overall survival (days) (except survivors)		
Median (range)	661 (297–1550)	506 (115–2087)

### Treatment of interstitial pneumonia in the case group

We identified 11 patients in the case group after exacerbation of interstitial pneumonia (Table 4). Corticosteroids were administered for interstitial pneumonia in 10 of the 11 patients. Of the ten patients, eight had improved interstitial pneumonia as a result of corticosteroids administration. One patient developed interstitial pneumonia after administration of the immunosuppressant tacrolimus in addition to corticosteroids. One patient died due to worsening lung cancer (disease progression) without improvement in interstitial pneumonia after corticosteroid administration.

**Table 4:** Treatment of interstitial pneumonia in the case group

Case	Age (years)	Sex	Immune checkpoint inhibitors	History of interstitial pneumonia	Radiological pattern of interstitial pneumonia	CTCAE grade of pneumonitis	Initial therapy of corticosteroids	Other treatment	Results of treatment
1	58	M	Pembrolizumab	Cause unknown; from the time of lung cancer diagnosis (idiopathic interstitial pneumonia^a^)	Usual interstitial pneumonia	Grade 3	Methylprednisolone sodium succinate drip infusion in vein 500 mg 3 days	Tacrolimus oral administration 4 mg	Oral corticosteroid administration continued while IV prednisolone was tapered Remission of interstitial pneumonia
2	74	M	Nivolumab	Nivolumab-induced interstitial pneumonia^b^	Organizing pneumonia pattern	Grade 3	Prednisolone sodium succinate drip infusion in vein 20 mg	None	Oral corticosteroid administration continued while IV prednisolone was tapered Remission of interstitial pneumonia
3	74	M	Atezolizumab	Cause unknown; from the time of lung cancer diagnosis (idiopathic interstitial pneumonia^a^)	Organizing pneumonia pattern	Grade 2	Oral prednisolone 30 mg (0.5 mg/kg)	None	Oral corticosteroid administration continued while IV prednisolone was tapered Remission of interstitial pneumonia
4	64	M	Nivolumab	Radiation pneumonitis	Organizing pneumonia pattern	Grade 3	Methylprednisolone sodium succinate drip infusion in vein 1000 mg 3 days	None	Oral corticosteroid administration continued while IV prednisolone was tapered Remission of interstitial pneumonia
5	75	M	Pembrolizumab	Pembrolizumab-induced interstitial pneumonia^b^	Organizing pneumonia pattern	Grade 3	Prednisolone sodium succinate drip infusion in vein 50 mg (1 mg/kg)	None	Oral corticosteroid administration continued while IV prednisolone was tapered Remission of interstitial pneumonia
6	68	M	Nivolumab	Nivolumab-induced interstitial pneumonia^b^	Organizing pneumonia pattern	Grade 4^c^	Methylprednisolone sodium succinatedrip infusion in vein 1000 mg 3 days	None	Prednisolone sodium succinate IV infusion 60 mg (1 mg/kg) death due to disease progression
7	69	M	Pembrolizumab	Pembrolizumab-induced interstitial pneumonia^b^	Organizing pneumonia pattern	Grade 1	Oral prednisolone 20 mg	None	Oral corticosteroid administration continued while IV prednisolone was tapered Remission of interstitial pneumonia
8	81	M	Nivolumab	Radiation pneumonitis	Nonspecific interstitial pneumonia	Grade 3	Prednisolone sodium succinate drip infusion in vein 60 mg (1 mg/kg)	None	Oral corticosteroid administration continued while IV prednisolone was tapered Remission of interstitial pneumonia
9	50	M	Nivolumab	Radiation pneumonitis	Organizing pneumonia pattern	Grade 2	Prednisolone sodium succinate drip infusion in vein 70 mg (1 mg/kg)	None	Oral corticosteroid administration continued while IV prednisolone was tapered Remission of interstitial pneumonia
10	71	M	Nivolumab	Radiation pneumonitis	Nonspecific interstitial pneumonia	Grade 3	Prednisolone sodium succinate drip infusion in vein 20 mg	None	Continue oral administration while tapering prednisolone interstitial pneumonia was remitted
11	76	F	Nivolumab	Radiation pneumonitis	Organizing pneumonia pattern	Grade 1	None (without hope for corticosteroids)	None	Oral corticosteroid administration continued while IV prednisolone was tapered Remission of interstitial pneumonia

*CTCAE* common terminology criteria for adverse events; *IV* intravenous

^a^Idiopathic interstitial pneumonia is an interstitial pneumonia of unknown cause

^b^Immune checkpoint inhibitor-induced interstitial pneumonia improved initially and then reoccurred when patients were re-challenged with immune checkpoint inhibitors

^c^Respiratory failure present

## Discussion

In this study, only idiopathic interstitial pneumonia was identified as a factor associated with a lower risk of developing interstitial pneumonia in NSCLC patients after ICI administration. There were no significant differences in other factors, such as smoking history, pulmonary emphysema, and chronic obstructive pulmonary disease. However, results suggest that administration of ICIs to patients with a history of idiopathic interstitial pneumonia might be associated with a lower risk of exacerbations of interstitial pneumonia than in those with a history of other types of NSCLC treatment-related interstitial pneumonia (i.e., immune-related pneumonitis and radiation pneumonitis). In addition, if there was a history of idiopathic interstitial pneumonia with a low risk of exacerbation of interstitial pneumonia, it would be possible to control the disease with corticosteroids after exacerbation. Clinical benefits such as prognosis prolongation due to the choice of ICIs might outweigh the disadvantages of exacerbation of interstitial pneumonia.

First, patients with a history of idiopathic interstitial pneumonia had significantly lower odds ratios for the exacerbation of interstitial pneumonia due to ICIs than those with a history of NSCLC treatment-related interstitial pneumonia. It has been suggested that ICI administration in patients with a history of idiopathic interstitial pneumonia might result in a lower risk of interstitial pneumonia relative to that of NSCLC treatment-related interstitial pneumonia. A history of interstitial pneumonia has been reported to be a risk factor for exacerbation of interstitial pneumonia [17]. However, the degree of risk due to different types of interstitial pneumonia (i.e., idiopathic interstitial pneumonia, immune-related pneumonitis, and radiation pneumonitis) has not been clarified. These results could contribute to elucidating the risk of interstitial pneumonia exacerbation due to different types of this condition.

Second, although this was a case–control study and simple comparisons were not possible, the median survival time in the case group was longer than that in the control group. It has been reported that patients with immune-related adverse events such as interstitial pneumonia have a better prognosis [18–20]. As previously reported, patients with a history of interstitial pneumonia might have clinical benefits due to prolonged survival facilitated by administering ICIs while paying attention to immune-related adverse events.

Thirdly, of the 11 patients whose interstitial pneumonia had worsened, 10 were in remission. Eight patients received corticosteroids, one received corticosteroids and tacrolimus, and one was withdrawn from ICIs. From this result, 10 of 11 patients (90.9%) who had a history of interstitial pneumonia were able to control their interstitial pneumonia. In a previous report, corticosteroids were administered to 207 patients who developed interstitial pneumonia after nivolumab administration for NSCLC [21]. Of these, 172 (83.1%) showed good responsiveness to corticosteroids. In this report, corticosteroids were administered to four patients with re-exacerbation of interstitial pneumonia, and three had a good response to this medicine. These results suggest that corticosteroids can control interstitial pneumonia even when the condition worsens in patients with a history of interstitial pneumonia worsen.

Based on these three points, idiopathic interstitial pneumonia might be associated with lower risk of interstitial pneumonia exacerbation than other types of NSCLC treatment-related interstitial pneumonia. In addition, even if interstitial pneumonia worsens, it might be possible to select ICI treatment because it can be controlled by corticosteroids.

In Japan, ICIs are recommended as first-line treatment if there are no gene mutations [3]. If there is a history of idiopathic interstitial pneumonia, there might be clinical benefits of ICIs administration, such as prognosis prolongation. For patients with a history of interstitial pneumonia, the clinical benefit of expanding treatment options with ICIs could be significant.

There are four limitations to this study.

First, individuals with a history of ICI-induced interstitial pneumonia were included in both the case (*n* = 4) and the control (*n* = 5) groups in this study. These patients were clinically diagnosed with “previous interstitial pneumonia,” which is an important consideration for ICI-based treatment. Since we conducted this study from the perspective of actual clinical practice, this can lead to serious bias.

Second, there was a selection bias only for patients treated in the university hospital. Without a well-established treatment system, ICIs might not be available for patients with a history of interstitial pneumonia at a high risk of exacerbation.

Third, unknown confounding factors could not be adjusted. This was a case–control study. Bias cannot be completely ruled out while selecting controls, because unknown confounders cannot be regulated. However, we considered the possibility of confounding in age and sex variables and matched these as covariates. In general, interstitial pneumonia is considered to be related to age, because physiological function declines with age. In addition, smoking history might be a confounding factor. An individual with a history of smoking is more likely to benefit from ICIs [5–7]. Smoking history is a risk factor for interstitial pneumonia [10, 15, 16]. However, we did not match patients based on smoking history due to the risk of overmatching. The presence or absence of a smoking history might have thus affected the results.

Fourth, the effects of concomitant anticancer drugs were not investigated in this study. It has been shown in previously reported clinical trials that the incidence of interstitial pneumonia is higher with combination therapy based on cytotoxic drugs (cisplatin, carboplatin, paclitaxel, and nanoparticle albumin-bound-paclitaxel) and ICIs than with monotherapy [22, 23]. In this study, 10 of 11 patients in the case group were treated with ICIs alone, not allowing for an analysis of the effects of the presence or absence of concomitant medications. In addition, the small number of 11 patients is also a limitation. Therefore, it is necessary to increase the number of cases further and examine the effects of concomitant anticancer drugs.

We conducted a case–control study of rare cases of interstitial pneumonia exacerbation after the administration of ICIs in patients with a history of interstitial pneumonia. Since rare cases were used as controls, the small number of 11 cases in the case group was unavoidable. However, due to the small number of cases, caution is required when interpreting the results. Therefore, based on the results of this study, it is necessary to consider the risk factors for interstitial pneumonia after increasing the number of target facilities and patients. Identifying the risk factors for interstitial pneumonia might lead to further clinical benefits for patients.

## Conclusions

Based on results of this study, ICIs could be a treatment option for patients with NSCLC who have a history of idiopathic interstitial pneumonia. In addition, even if interstitial pneumonia worsens, corticosteroids might be able to control it. Therefore, the selection of ICIs for patients with NSCLC with a history of idiopathic interstitial pneumonia could have clinical benefits.

## Data Availability

All relevant data are within the manuscript and the supporting information files.
